# Methyl 4-anilino-3-nitro­benzoate

**DOI:** 10.1107/S1600536809018923

**Published:** 2009-05-23

**Authors:** Hao-Yuan Li, Yong-Zhong Wu, Bo-Nian Liu, Shi-Gui Tang, Cheng Guo

**Affiliations:** a College of Biotechnology and Pharmaceutical Engineering, Nanjing University of Technology, Xinmofan Road No. 5 Nanjing, Nanjing 210009, People’s Republic of China; b Department of Applied Chemistry, Nanjing College of Chemical Technology, Geguan Road No. 625 Dachang District Nanjing, Nanjing 210048, People’s Republic of China; c College of Science, Nanjing University of Technology, Xinmofan Road No. 5 Nanjing, Nanjing 210009, People’s Republic of China

## Abstract

In the mol­ecule of the title compound, C_14_H_12_N_2_O_4_, the aromatic rings are oriented at a dihedral angle of 51.50 (4)°. An intra­molecular N—H⋯O inter­action results in the formation of a six-membered ring having an envelope conformation. In the crystal structure, inter­molecular N—H⋯O inter­actions link the mol­ecules into centrosymmetric dimers. π–π contacts between the benzene rings [centroid–centroid distance = 3.708 (1) Å] may further stabilize the structure.

## Related literature

For bond-length data, see: Allen *et al.* (1987 ▶). For the synthesis, see: Schelz (1978 ▶).
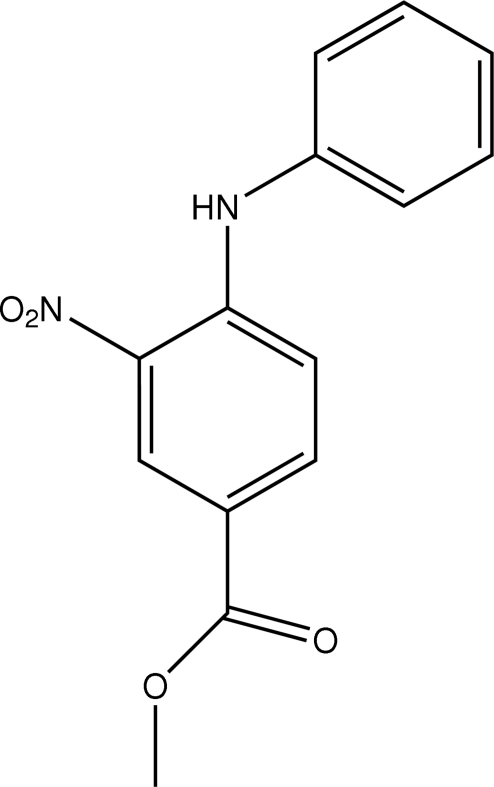


## Experimental

### 

#### Crystal data

C_14_H_12_N_2_O_4_
*M* *_r_* = 272.26
Monoclinic, 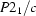

*a* = 11.641 (2) Å
*b* = 16.349 (3) Å
*c* = 7.2490 (14) Å
β = 107.50 (3)°
*V* = 1315.8 (5) Å^3^
*Z* = 4
Mo *K*α radiation
μ = 0.10 mm^−1^
*T* = 294 K
0.30 × 0.20 × 0.10 mm

#### Data collection

Enraf–Nonius CAD-4 diffractometer
Absorption correction: ψ scan (North *et al.*, 1968 ▶) *T*_min_ = 0.970, *T*_max_ = 0.990
2569 measured reflections
2367 independent reflections
1335 reflections with *I* > 2σ(*I*)
*R*_int_ = 0.026
3 standard reflections frequency: 120 min intensity decay: 1%

#### Refinement

*R*[*F* ^2^ > 2σ(*F* ^2^)] = 0.066
*wR*(*F* ^2^) = 0.178
*S* = 1.00
2367 reflections
175 parameters
H-atom parameters constrained
Δρ_max_ = 0.33 e Å^−3^
Δρ_min_ = −0.44 e Å^−3^

### 

Data collection: *CAD-4 Software* (Enraf–Nonius, 1989 ▶); cell refinement: *CAD-4 Software*; data reduction: *XCAD4* (Harms & Wocadlo, 1995 ▶); program(s) used to solve structure: *SHELXS97* (Sheldrick, 2008 ▶); program(s) used to refine structure: *SHELXL97* (Sheldrick, 2008 ▶); molecular graphics: *ORTEP-3 for Windows* (Farrugia, 1997 ▶) and *PLATON* (Spek, 2009 ▶); software used to prepare material for publication: *SHELXL97*.

## Supplementary Material

Crystal structure: contains datablocks global, I. DOI: 10.1107/S1600536809018923/hk2689sup1.cif

Structure factors: contains datablocks I. DOI: 10.1107/S1600536809018923/hk2689Isup2.hkl

Additional supplementary materials:  crystallographic information; 3D view; checkCIF report

## Figures and Tables

**Table 1:** Hydrogen-bond geometry (Å, °)

*D*—H⋯*A*	*D*—H	H⋯*A*	*D*⋯*A*	*D*—H⋯*A*
N1—H1*A*⋯O1	0.86	2.01	2.650 (4)	130
N1—H1*A*⋯O1^i^	0.86	2.53	3.314 (4)	152

Symmetry code: (i) 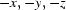.

**Fig. 1.**
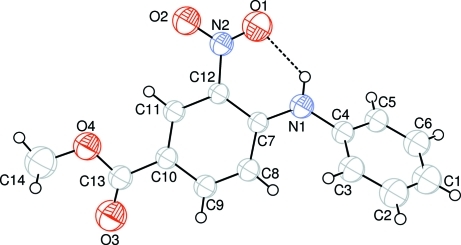
The molecular structure of the title molecule, with the atom-numbering scheme. Hydrogen bond is shown as dashed line.

**Fig. 2.**
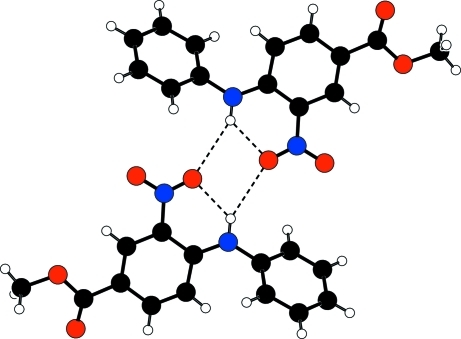
A partial packing diagram of the title compound. Hydrogen bonds are shown as dashed lines.

## supplementary crystallographic information

### Comment

Some derivatives of benzoic acid are important chemical materials. We report herein the crystal structure of the title compound.

In the molecule of the title compound (Fig 1), the bond lengths (Allen *et al.*,  1987) and angles are within normal ranges. Rings A (C1-C6) and B (C7-C12) are, of course, planar and they are oriented at a dihedral angle of A/B = 51.50 (4)°. Intramolecular N-H···O interaction (Table 1) results in the formation of a six-membered ring C (O1/N1/N2/C7/C12/H1A) having envelope conformation with atom O1 displaced by 0.125 (4) Å from the plane of the other ring atoms.

In the crystal structure, intra- and intermolecular N-H···O interactions (Table 1) link the molecules into centrosymmetric dimers (Fig. 2), in which they may be effective in the stabilization of the structure. The π–π contact between the benzene rings, Cg2—Cg2^i^ [symmetry code: (i) x, 1/2 - y, z - 1/2, where Cg2 is centroid of the ring B (C7-C12)] may further stabilize the structure, with centroid-centroid distance of 3.708 (1) Å.

### Experimental

For the preparation of the title compound, methyl 4-chloro-3-nitrobenzoate (5.0 g, 23 mmol) was heated in distilled aniline (10 ml) for 18 h at 393 K. After the reaction was completed, ethanol (50 ml) was added, at room temperature. The yellow precipitate was washed with cold ethanol (2 × 20 ml), and then dried (yield; 4.7 g). Crystals suitable for X-ray analysis were obtained by slow evaporation of an ethanol solution.

### Refinement

H atoms were positioned geometrically, with N-H = 0.86 Å (for NH) and C-H = 0.93 and 0.96 Å for aromatic and methyl H, respectively, and constrained to ride on their parent atoms, with U_iso_(H) = xU_eq_(C,N), where x = 1.5 for methyl H and x = 1.2 for all other H atoms.

### Crystal data

**Table:**

C_14_H_12_N_2_O_4_	*F*(000) = 568
*M**_r_* = 272.26	*D*_x_ = 1.374 Mg m^−^^3^
Monoclinic, *P*2_1_/*c*	Mo *K*α radiation, λ = 0.71073 Å
Hall symbol: -P 2ybc	Cell parameters from 25 reflections
*a* = 11.641 (2) Å	θ = 10–12°
*b* = 16.349 (3) Å	µ = 0.10 mm^−^^1^
*c* = 7.2490 (14) Å	*T* = 294 K
β = 107.50 (3)°	Block, colorless
*V* = 1315.8 (5) Å^3^	0.30 × 0.20 × 0.10 mm
*Z* = 4	

### Data collection

**Table:**

Enraf–Nonius CAD-4 diffractometer	1335 reflections with *I* > 2σ(*I*)
Radiation source: fine-focus sealed tube	*R*_int_ = 0.026
graphite	θ_max_ = 25.2°, θ_min_ = 1.8°
ω/2θ scans	*h* = −13→13
Absorption correction: ψ scan (North *et al.*, 1968)	*k* = −19→0
*T*_min_ = 0.970, *T*_max_ = 0.990	*l* = 0→8
2569 measured reflections	3 standard reflections every 120 min
2367 independent reflections	intensity decay: 1%

### Refinement

**Table:**

Refinement on *F*^2^	Secondary atom site location: difference Fourier map
Least-squares matrix: full	Hydrogen site location: inferred from neighbouring sites
*R*[*F*^2^ > 2σ(*F*^2^)] = 0.066	H-atom parameters constrained
*wR*(*F*^2^) = 0.178	*w* = 1/[σ^2^(*F*_o_^2^) + (0.08*P*)^2^ + 0.4*P*] where *P* = (*F*_o_^2^ + 2*F*_c_^2^)/3
*S* = 1.00	(Δ/σ)_max_ < 0.001
2367 reflections	Δρ_max_ = 0.33 e Å^−^^3^
175 parameters	Δρ_min_ = −0.44 e Å^−^^3^
Primary atom site location: structure-invariant direct methods	

### Special details

**Table:**

Geometry. All e.s.d.'s (except the e.s.d. in the dihedral angle between two l.s. planes) are estimated using the full covariance matrix. The cell e.s.d.'s are taken into account individually in the estimation of e.s.d.'s in distances, angles and torsion angles; correlations between e.s.d.'s in cell parameters are only used when they are defined by crystal symmetry. An approximate (isotropic) treatment of cell e.s.d.'s is used for estimating e.s.d.'s involving l.s. planes.
Refinement. Refinement of *F*^2^ against ALL reflections. The weighted *R*-factor *wR* and goodness of fit *S* are based on *F*^2^, conventional *R*-factors *R* are based on *F*, with *F* set to zero for negative *F*^2^. The threshold expression of *F*^2^ > σ(*F*^2^) is used only for calculating *R*-factors(gt) *etc*. and is not relevant to the choice of reflections for refinement. *R*-factors based on *F*^2^ are statistically about twice as large as those based on *F*, and *R*- factors based on ALL data will be even larger.

### Fractional atomic coordinates and isotropic or equivalent isotropic displacement parameters (Å^2^)

**Table:**

	*x*	*y*	*z*	*U*_iso_*/*U*_eq_	
O1	0.0837 (2)	0.05896 (17)	0.1085 (4)	0.078	
O2	0.2328 (2)	0.11328 (16)	0.2971 (4)	0.0677 (9)	
O3	0.1608 (3)	0.48217 (18)	0.1788 (5)	0.0848 (10)	
O4	0.2843 (2)	0.39247 (16)	0.3690 (4)	0.0648 (8)	
N1	−0.1155 (2)	0.14101 (18)	−0.0669 (4)	0.0501 (8)	
H1A	−0.0836	0.0932	−0.0446	0.060*	
N2	0.1335 (3)	0.11733 (17)	0.1801 (5)	0.0479 (8)	
C1	−0.4811 (4)	0.1408 (3)	−0.3820 (7)	0.0754 (14)	
H1B	−0.5621	0.1397	−0.4538	0.091*	
C2	−0.4423 (4)	0.1887 (3)	−0.2185 (7)	0.0721 (13)	
H2A	−0.4973	0.2200	−0.1789	0.087*	
C3	−0.3218 (3)	0.1902 (3)	−0.1139 (6)	0.0575 (11)	
H3A	−0.2959	0.2222	−0.0031	0.069*	
C4	−0.2393 (3)	0.1441 (2)	−0.1731 (5)	0.0440 (9)	
C5	−0.2794 (3)	0.0959 (2)	−0.3384 (5)	0.0504 (10)	
H5A	−0.2254	0.0647	−0.3808	0.061*	
C6	−0.3991 (4)	0.0951 (3)	−0.4367 (6)	0.0638 (12)	
H6A	−0.4259	0.0621	−0.5457	0.077*	
C7	−0.0420 (3)	0.2050 (2)	0.0031 (5)	0.0378 (8)	
C8	−0.0795 (3)	0.2868 (2)	−0.0439 (5)	0.0460 (9)	
H8A	−0.1567	0.2963	−0.1260	0.055*	
C9	−0.0075 (3)	0.3514 (2)	0.0258 (5)	0.0465 (9)	
H9A	−0.0363	0.4039	−0.0101	0.056*	
C10	0.1095 (3)	0.3414 (2)	0.1510 (5)	0.0431 (9)	
C11	0.1507 (3)	0.2631 (2)	0.1962 (5)	0.0416 (9)	
H11A	0.2284	0.2549	0.2777	0.050*	
C12	0.0786 (3)	0.1958 (2)	0.1226 (5)	0.0385 (8)	
C13	0.1830 (4)	0.4131 (2)	0.2287 (6)	0.0509 (10)	
C14	0.3604 (4)	0.4579 (3)	0.4546 (7)	0.0886 (16)	
H14A	0.4293	0.4372	0.5528	0.133*	
H14B	0.3174	0.4954	0.5116	0.133*	
H14C	0.3865	0.4858	0.3576	0.133*	

### Atomic displacement parameters (Å^2^)

**Table:**

	*U*^11^	*U*^22^	*U*^33^	*U*^12^	*U*^13^	*U*^23^
O1	0.064	0.060	0.091	0.006	−0.005	0.000
O2	0.0506 (16)	0.0573 (18)	0.075 (2)	0.0069 (14)	−0.0122 (15)	−0.0036 (15)
O3	0.090 (2)	0.0424 (18)	0.108 (3)	−0.0121 (16)	0.009 (2)	0.0030 (17)
O4	0.0496 (16)	0.0559 (18)	0.078 (2)	−0.0124 (13)	0.0026 (15)	−0.0116 (15)
N1	0.0426 (17)	0.0421 (17)	0.056 (2)	−0.0071 (14)	0.0013 (15)	−0.0022 (15)
N2	0.0406 (17)	0.0336 (16)	0.058 (2)	−0.0125 (13)	−0.0025 (16)	−0.0141 (14)
C1	0.045 (2)	0.082 (3)	0.082 (3)	−0.021 (2)	−0.007 (2)	0.010 (3)
C2	0.044 (2)	0.086 (3)	0.086 (4)	−0.006 (2)	0.017 (2)	−0.007 (3)
C3	0.044 (2)	0.071 (3)	0.053 (3)	−0.010 (2)	0.0090 (19)	−0.012 (2)
C4	0.040 (2)	0.044 (2)	0.042 (2)	−0.0085 (17)	0.0045 (17)	0.0034 (17)
C5	0.053 (2)	0.046 (2)	0.046 (2)	−0.0152 (18)	0.0058 (19)	−0.0013 (18)
C6	0.061 (3)	0.071 (3)	0.049 (3)	−0.021 (2)	0.000 (2)	−0.001 (2)
C7	0.0369 (18)	0.043 (2)	0.0349 (19)	−0.0075 (16)	0.0124 (15)	−0.0044 (16)
C8	0.039 (2)	0.054 (2)	0.043 (2)	−0.0007 (17)	0.0093 (17)	0.0032 (18)
C9	0.047 (2)	0.038 (2)	0.054 (2)	0.0001 (17)	0.0157 (18)	0.0050 (18)
C10	0.047 (2)	0.044 (2)	0.042 (2)	−0.0069 (17)	0.0171 (17)	−0.0040 (17)
C11	0.0335 (18)	0.051 (2)	0.039 (2)	−0.0018 (16)	0.0094 (16)	−0.0036 (17)
C12	0.0343 (18)	0.0368 (19)	0.044 (2)	−0.0010 (15)	0.0115 (16)	−0.0030 (16)
C13	0.058 (2)	0.042 (2)	0.057 (3)	−0.0085 (19)	0.022 (2)	−0.0058 (19)
C14	0.068 (3)	0.090 (4)	0.096 (4)	−0.037 (3)	0.006 (3)	−0.029 (3)

### Geometric parameters (Å, °)

**Table:**

O1—N2	1.155 (3)	C5—C6	1.361 (5)
O2—N2	1.213 (3)	C5—H5A	0.9300
O3—C13	1.190 (5)	C6—H6A	0.9300
O4—C13	1.348 (4)	C7—C8	1.416 (5)
O4—C14	1.409 (5)	C7—C12	1.419 (4)
N1—C7	1.349 (4)	C8—C9	1.348 (5)
N1—C4	1.416 (4)	C8—H8A	0.9300
N1—H1A	0.8600	C9—C10	1.401 (5)
N2—C12	1.438 (4)	C9—H9A	0.9300
C1—C6	1.361 (6)	C10—C11	1.372 (5)
C1—C2	1.378 (6)	C10—C13	1.461 (5)
C1—H1B	0.9300	C11—C12	1.389 (4)
C2—C3	1.379 (5)	C11—H11A	0.9300
C2—H2A	0.9300	C14—H14A	0.9600
C3—C4	1.387 (5)	C14—H14B	0.9600
C3—H3A	0.9300	C14—H14C	0.9600
C4—C5	1.392 (5)		
			
C13—O4—C14	115.7 (3)	N1—C7—C12	123.1 (3)
C7—N1—C4	127.1 (3)	C8—C7—C12	115.0 (3)
C7—N1—H1A	116.5	C9—C8—C7	122.6 (3)
C4—N1—H1A	116.5	C9—C8—H8A	118.7
O1—N2—O2	120.9 (3)	C7—C8—H8A	118.7
O1—N2—C12	119.2 (3)	C8—C9—C10	121.6 (3)
O2—N2—C12	119.9 (3)	C8—C9—H9A	119.2
C6—C1—C2	119.0 (4)	C10—C9—H9A	119.2
C6—C1—H1B	120.5	C11—C10—C9	117.8 (3)
C2—C1—H1B	120.5	C11—C10—C13	122.3 (3)
C1—C2—C3	119.9 (4)	C9—C10—C13	119.9 (3)
C1—C2—H2A	120.1	C10—C11—C12	121.3 (3)
C3—C2—H2A	120.1	C10—C11—H11A	119.3
C2—C3—C4	120.3 (4)	C12—C11—H11A	119.3
C2—C3—H3A	119.8	C11—C12—C7	121.5 (3)
C4—C3—H3A	119.8	C11—C12—N2	115.5 (3)
C3—C4—C5	119.3 (3)	C7—C12—N2	122.9 (3)
C3—C4—N1	122.6 (3)	O3—C13—O4	121.9 (4)
C5—C4—N1	118.1 (3)	O3—C13—C10	126.6 (4)
C6—C5—C4	118.7 (4)	O4—C13—C10	111.6 (3)
C6—C5—H5A	120.6	O4—C14—H14A	109.5
C4—C5—H5A	120.6	O4—C14—H14B	109.5
C5—C6—C1	122.7 (4)	H14A—C14—H14B	109.5
C5—C6—H6A	118.7	O4—C14—H14C	109.5
C1—C6—H6A	118.7	H14A—C14—H14C	109.5
N1—C7—C8	121.8 (3)	H14B—C14—H14C	109.5
			
C6—C1—C2—C3	0.3 (7)	C13—C10—C11—C12	179.1 (4)
C1—C2—C3—C4	0.5 (7)	C10—C11—C12—C7	−2.3 (5)
C2—C3—C4—C5	−0.5 (6)	C10—C11—C12—N2	179.4 (3)
C2—C3—C4—N1	−177.5 (4)	N1—C7—C12—C11	−178.3 (3)
C7—N1—C4—C3	−48.9 (5)	C8—C7—C12—C11	3.8 (5)
C7—N1—C4—C5	134.0 (4)	N1—C7—C12—N2	−0.1 (5)
C3—C4—C5—C6	−0.3 (6)	C8—C7—C12—N2	−178.0 (3)
N1—C4—C5—C6	176.9 (3)	O1—N2—C12—C11	−171.9 (4)
C4—C5—C6—C1	1.2 (6)	O2—N2—C12—C11	5.4 (5)
C2—C1—C6—C5	−1.2 (7)	O1—N2—C12—C7	9.8 (6)
C4—N1—C7—C8	−7.9 (6)	O2—N2—C12—C7	−172.9 (3)
C4—N1—C7—C12	174.4 (3)	C14—O4—C13—O3	1.2 (6)
N1—C7—C8—C9	179.6 (4)	C14—O4—C13—C10	−179.3 (4)
C12—C7—C8—C9	−2.5 (5)	C11—C10—C13—O3	169.8 (4)
C7—C8—C9—C10	−0.3 (6)	C9—C10—C13—O3	−10.4 (6)
C8—C9—C10—C11	2.1 (6)	C11—C10—C13—O4	−9.8 (5)
C8—C9—C10—C13	−177.8 (3)	C9—C10—C13—O4	170.1 (3)
C9—C10—C11—C12	−0.8 (5)		

### Hydrogen-bond geometry (Å, °)

**Table:**

*D*—H···*A*	*D*—H	H···*A*	*D*···*A*	*D*—H···*A*
N1—H1A···O1	0.86	2.01	2.650 (4)	130
N1—H1A···O1^i^	0.86	2.53	3.314 (4)	152

Symmetry codes: (i) −*x*, −*y*, −*z*.
